# The Fit for School health outcome study - a longitudinal survey to assess health impacts of an integrated school health programme in the Philippines

**DOI:** 10.1186/1471-2458-13-256

**Published:** 2013-03-21

**Authors:** Bella Monse, Habib Benzian, Ella Naliponguit, Vincente Belizario, Alexander Schratz, Wim van Palenstein Helderman

**Affiliations:** 1 Deutsche Gesellschaft für Internationale Zusammenarbeit (GIZ) GmbH, GIZ Office Manila, PDCP Bank Centre, V.A. Rufino cor. L.P. Leviste Str, Makati, Metro Manila, Philippines; 2 Senior Advisor, Fit for School Inc./The Health Bureau Ltd., The Little Barn, Haversham Manor, MK19 7DZ, Haversham, Milton Keynes, UK; 3 Health and Nutrition Department, Department of Education, Manila, Philippines; 4 National Institutes of Health, University of the Philippines, 623 Pedro Gil Street, Ermita 1000, Metro Manila, Philippines; 5 Fit for School Inc., Makati Office, PDCP Bank Centre, V.A. Rufino cor. L.P. Leviste Str, Makati, Metro Manila, Philippines; 6 Dental Health International Nederland(DHIN), Korte Linschoten, OZ 14, 3461CG, Linschoten, Netherlands

**Keywords:** School health, Soil-transmitted helminth infection, Child growth, Dental caries, Hand washing, Toothbrushing, Deworming

## Abstract

**Background:**

Child health in many low- and middle-income countries lags behind international goals and affects children’s education, well-being, and general development. Large-scale school health programmes can be effective in reducing preventable diseases through cost-effective interventions. This paper outlines the baseline and 1-year results of a longitudinal health study assessing the impact of the Fit for School Programme in the Philippines.

**Methods:**

A longitudinal 4-year cohort study was conducted in the province of Camiguin, Mindanao (experimental group); an external concurrent control group was studied in Gingoog, Mindanao. The study has three experimental groups: group 1—daily handwashing with soap, daily brushing with fluoride toothpaste, biannual deworming with 400 mg albendazole (Essential Health Care Program [EHCP]); group 2—EHCP plus twice-a-year access to school-based Oral Urgent Treatment; group 3—EHCP plus weekly toothbrushing with high-fluoride concentration gel. A non-concurrent internal control group was also included. Baseline data on anthropometric indicators to calculate body mass index (BMI), soil-transmitted helminths (STH) infection in stool samples, and dental caries were collected in August 2009 and August 2010. Data were analysed to assess validity of the control group design, baseline, and 1-year results.

**Results:**

In the cohort study, 412 children were examined at baseline and 341 1 year after intervention. The baseline results were in line with national averages for STH infection, BMI, and dental caries in group 1 and the control groups. Children lost to follow-up had similar baseline characteristics in the experimental and control groups. After 1 year, group 1 showed a significantly higher increase in mean BMI and lower prevalence of moderate to heavy STH infection than the external concurrent control group. The increases in caries and dental infections were reduced but not statistically significant. The results for groups 2 and 3 will be reported separately.

**Conclusions:**

Despite the short 1-year observation period, the study found a reduction in the prevalence of moderate to heavy STH infections, a rise in mean BMI, and a (statistically non-significant) reduction in dental caries and infections. The study design proved functional in actual field conditions. Critical aspects affecting the validity of cohort studies are analysed and discussed.

**Trial registration:**

DRKS00003431 WHO Universal Trial Number U1111-1126-0718

## Background

The health and education of children are a public good that lies at the core of government policies and programmes. The Millennium Development Goals have encouraged significant resource allocation to these two sectors, which are closely related to long-term poverty reduction and development, and much progress has been made. Still, many low- and middle-income countries are unlikely to reach the health- and education-related targets to which they have committed themselves. The 2011 United Nations Millennium Development Goals Report clearly states, “Despite real progress, we are failing to reach the most vulnerable” [1].

As a low-middle-income country, the Philippines is one such case: there are persistent high levels of preventable diseases among children in addition to poor primary education indicators [2]. Ill health is the main reason for school absenteeism and dropouts (about 40% of dropouts are due to illness; toothache is the most common reason for absenteeism) [3-5]. Filipino children mainly suffer from a few widespread diseases: diarrhoea, pneumonia, and respiratory infections are the leading cause of death (82,000 children in the 5- to 12-year age group die from these every year), and 54% of children are infested with soil-transmitted helminths (STH) [6-8]. One-third of children are stunted, and 17% have a below-normal body mass index (BMI) [9]. The prevalence of dental caries is extreme: more than 97% of 6-year-old children suffer from tooth decay; it usually goes untreated, which leads to very high rates of dental infection (85% of 6-year olds show such signs) [10].

### Essential health care programme in the Philippines

School health programmes have the potential to contribute significantly to preventing and controlling key diseases in children, particularly if the programmes are able to systematically reach mass numbers of children, thereby producing benefits for both health and education [11,12]. The Philippine Essential Health Care Program (EHCP), also known as the Fit for School Programme, aims to address some of the major diseases that affect the Filipino child population through simple, evidence-based, integrated approaches [13-15]. The EHCP currently targets more than 2 million children in the Philippines. The EHCP was developed by the Health and Nutrition Center of the Department of Education Central Office and the Philippine National Institutes of Health. However, it operates in the greater context of official development assistance linking Philippine and European universities. As a result, the EHCP was developed in close cooperation with the following organizations: the German Development Cooperation (GIZ); the World Health Organization (WHO) Collaborating Center for Prevention of Oral Diseases at the University of Jena in Germany; the former WHO Collaborating Center on Oral Health Care and Future Scenarios in Nijmegen, the Netherlands; and Xavier University in Cagayan de Oro, Philippines. the EHCP is part of a bigger context of official development assistance linking in Philippine and European universities.

The EHCP aims to improve child health and development by institutionalization of three preventive interventions within public elementary schools in the Philippines: daily supervised handwashing with soap and clean water; daily supervised brushing with fluoride toothpaste; and biannual deworming via mass drug administration. This innovative approach is conceptually based on the Fit for School Action Framework which outlines the principles of simplicity, scalability, and sustainability [16]. The approach was awarded by the Worldbank, UNDP and the WHO for innovation in global health in 2009.

### Fit for School health outcome study

The Fit for School Health Outcome Study (FITHOS) is a longitudinal cohort survey whose objective is to provide data on the impact of established interventions that are part of the EHCP and implemented in schools using the manpower and financial resources of the government school system. The FITHOS is financed by GIZ and conducted by local institutions (such as the regional Department of Education Health and Nutrition Unit in Cagayan de Oro). This study is part of efforts to improve local capacity in applied research, and it aims to provide evidence for informed management and policy decisions.

The FITHOS is a large study, and it includes a range of health and health-related parameters, e.g. quality of life, school days lost because of illness, and education performance. Furthermore, two interventions toward controlling dental caries are also part of the FITHOS in order to assess their impact and feasibility in the school context. The details of those dental interventions, however, are not presented in this paper owing to lack of space and will be reported in a separate publication. We will introduce and discuss the methodology of this community trial and briefly present the baseline and 1-year data as the direct health outcomes of the three integrated interventions of the EHCP.

## Methods

### Study design

**Figure 1:**
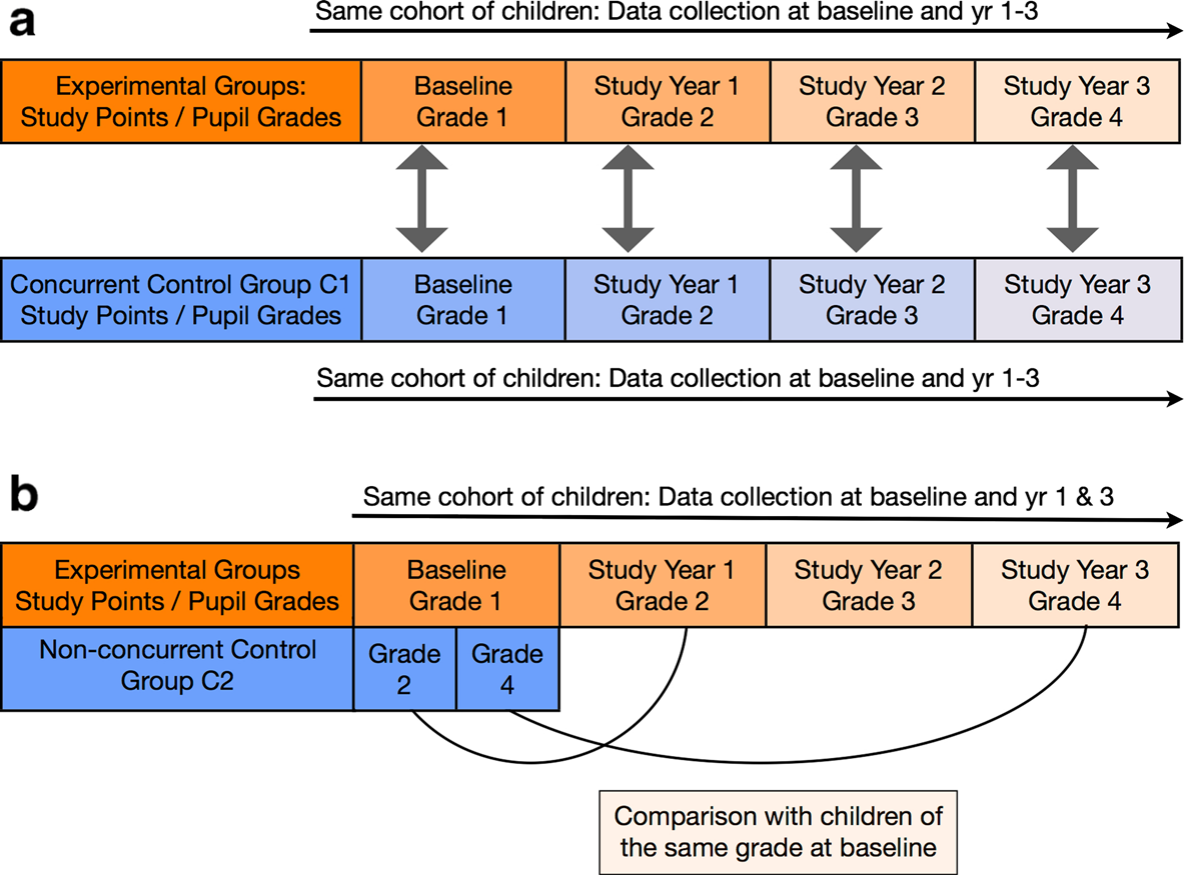
**Longitudinal cohort design with an external concurrent control group and an internal non-concurrent control group. a**. Data collection & comparisons for concurrent external control group. **b**. Data collection & comparisons for non-concurrent internal control group.

The study applies a longitudinal cohort design over a period of 4 years with an external concurrent control group and an internal (within-school) non-concurrent control group (Figure 1). The study started in 2009 with baseline data collection among first-grade students (6–7 years old) of public elementary schools on the island province of Camiguin. Subsequent annual data collection has been scheduled for August of every following year up to 2013 (with optional extension until 2014). Camiguin was selected because of its comparably low migration rate and stable socioeconomic indicators. Based on a local government decision to implement the EHCP in the entire province, it was not possible to assign a control group with children not participating in the EHCP in that province. An external concurrent control group was therefore identified in Gingoog, Misamis Oriental, Northern Mindanao. The local government authorities of Gingoog had no intention of implementing the EHCP during the study period, and the local child population was similar to that in Camiguin in terms of health and socioeconomic characteristics.

Public elementary schools participating in the study (experimental and control groups) were selected based on two criteria: (1) location along a highway or no more than 1 kilometre from a highway; and (2) no problems related to law and order in the surrounding community.

The calculation of the study cohort size was based on the following assumptions: a 50% reduction in the prevalence of moderate to heavy STH infections; an assumed caries prevented fraction (using the Decayed-, Missing-, Filled Surfaces Index [DMFS] control – caries DMFS intervention / caries DMFS control × 100%) of 30% in permanent first molars over the study period; and an anticipated dropout rate of 40% during that period. Though a group of around 100 children would have allowed for statistical power of 80% and 95% confidence intervals, a group size of 200 children at baseline was considered safer and sufficient to cover all eventualities.

For this sample size, four public elementary schools in Camiguin (of a total of 56 schools on the island) were randomly assigned to the EHCP experimental group based on the above two criteria. The Department of Education Health and Nutrition Unit selected three schools in Gingoog as concurrent control schools (not participating in the EHCP). Children with systemic medical conditions and other chronic infectious diseases, such as tuberculosis, were excluded from the study.

### Experimental groups

The study protocol defined three experimental groups, as detailed below. However, this paper reports only on the baseline and 1-year results of experimental group 1. Experimental groups 2 and 3 were used to test additional interventions in controlling caries, and those results will be presented in a separate paper.

#### Experimental group 1

This group participates in the EHCP, which includes the following: daily supervised handwashing with soap and clean water (as a scheduled group activity); daily supervised brushing with a fluoride toothpaste (0.3 ml; 1,450 ppm free available fluoride, scheduled group activity); and biannual deworming with a single dose of albendazole (400 mg) as a mass drug administration at school. These interventions are implemented by education staff (teachers for daily tasks, school health nurses of the Department of Education for orientation and supervision) as defined in the EHCP protocol.

#### Experimental group 2

The second experimental group participates in the EHCP (like experimental group 1), and it has access to Oral Urgent Treatment (as defined by WHO), in which treatment is offered twice a year at every school for children suffering from toothache due to advanced dental caries. This on-demand treatment includes tooth extractions, drainage of abscesses, and drug administration in selected cases [17].

#### Experimental group 3

The third experimental group participates in the EHCP (like experimental group 1), and it uses a high-concentration fluoride gel (Elmex Gel®, Gaba GmbH Lörrach, Germany in a dispenser, releasing 0.3 ml per usage; total fluoride concentration 12,500 ppm) once a week instead of regular toothpaste. Children use normal toothpaste on the other 4 school days. The fluoride gel is applied on the toothbrush and children brush with it after normal brushing for 2 minutes.

### Control groups

#### External concurrent control group (Group C1)

Located in Gingoog City, this cohort of children receives a standard health education programme as defined by the Department of Education. It consists of an annual physical examination, biannual deworming carried out by school nurses, the distribution of a single (10-ml) commercial toothpaste sachet, a toothbrush, and an oral health message at the beginning of the school year, and health education as part of the regular school curriculum (Figure 1a).

#### Internal non-concurrent control group (Group C2)

This group consists of children in the participating EHCP schools in grades 2 and 4 whose data were collected during the baseline assessment. Since the intervention child cohort from the baseline moves from grade 1 to grade 5 during the study period, the data from children in grades 2 and grade 4 are used to serve as a control for the experimental group (Figure 1b).

### Examiner training and calibration

One week before the start of the study, a 2-day examiner training session was conducted. School nurses were trained in standardized collection of stool samples, obtaining height and weight data, and regular calibration of weighing scales. Oral examinations were conducted by three dentists after 2 days of theoretical and clinical training in the diagnosis methodology. One of the dentists had participated in the last national oral health survey and was used as the gold standard [10]. To assess reproducibility, 7.5% of children were examined twice. All examiners were blind to the different groups. Experienced examiners used a standard questionnaire to collect personal and socio-demographic data.

### Anthropometric measures

Trained school nurses took all measurements according to standard guidelines [18]. Children removed their shoes and stood upright for measurement of height using a portable stadiometer (Seca, Hamburg, Germany) to the nearest 0.5 cm. Their weight was measured with a portable weighing scale to the nearest 0.1 kg (Detecto® Cardinal Scale Manufacturing Co., Webb City, USA). Generally the children were only lightly dressed, so no adjustments were made for clothing. Anthropometric measuring equipment was re-calibrated at the beginning of each day and after every 10th child using a plastic bag filled with 6 kg of sand. Height and weight were used to compute BMI for age [19,20]. The BMI of each child was calculated as the body weight in kilos divided by the height in metres squared—weight (kg)/height (m^2^). The results were grouped as normal, below-, and above-normal BMI according to the sex- and age-related cut-off points of Cole et al. [21].

### Parasitological examination

Each child submitted a stool sample, which was labelled, coded, and sent daily (by courier) to the laboratory of the National Institutes of Health, University of the Philippines, in Manila. Samples were examined to determine the prevalence and intensity of STH infection using the Kato-Katz method [22]. Cut-off points defined by WHO were used to classify light-, moderate-, and heavy-intensity infections [23]. For quality control of parasitological examinations, 10% of all slides were randomly selected and re-examined by a reference microscopist.

### Oral examination

An assessment was made of the prevalence of dental caries and oral infections. The children brushed their teeth before the examination. Oral examinations were performed in the schoolyard in the open air, with children lying supine on benches taken from a classroom. Mouth mirrors (lighted mouth mirror Mirrorlight™, Kudos, Hong Kong) and a CPI ball-end probe were used as examination tools to score caries according to the WHO basic methods for epidemiological oral health surveys Decayed-, Missing-, Filled Index (DMF) [24]. Teeth with early stages of caries, but where the ball-end probe was unable to enter, were not scored as caries and excluded from analysis. Oral infections were recorded according to criteria for the PUFA index [25] : PUFA measures the consequences of untreated dental caries, such as open pulp, ulceration, fistula and abscess, with PUFA values representing permanent teeth and pufa values primary teeth.

### Statistical analysis

The data were analysed using SAS 9.1 software (SAS Institute, USA). Inter-examiner reproducibility for caries score and dental infection (PUFA) at baseline and at follow-up examinations was calculated using kappa statistics. The reproducibility of parasitological examinations was assessed with sensitivity and specificity values. The outcome variables in the longitudinal design are presented as mean increments: percentages for prevalence data and means for caries score (DMFS and PUFA) and BMI (Table 1). The outcome variables in the cross-sectional design are presented as means: percentages for prevalence data and means for caries scores and BMI (Table 2). For differences between mean (increment) data, Student’s *t* test was applied. For differences between percentages of increment data below normal BMI, the chi-square test was applied with 2 × 4 cells, where each group was divided in: normal remains normal, below normal remains below normal, normal moves to below normal, and below normal moves to normal. For differences between percentages of increment data of moderate to heavy STH infections the chi-square test was applied with 2 × 4 cells, where each group was divided in: heavy remains heavy, low remains low, heavy moves to low, and low moves to heavy.

**Table 1:** Mean (± se) baseline data, 1-year data, and incremental data for experimental group and the external concurrent control group

**Indicators**	**Experimental group 1**			**External concurrent control group (C1)**			**Difference between increments (p-value)**
	n=168			n=173			
	Baseline	1-year	increment	Baseline	1-year	increment	
Mean BMI	14.70 (0.11)	14.88 (0.13)	0.18 (0.06)	14.65 (0.11)	14.62 (0.11)	−0.03 (0.05)	Student-t p<0.01
Prevalence of below normal BMI	29.2% (3.5)	27.8% (3.5)	−1.4%	31.8% (3.5)	37.6% (3.7)	5.8%	Chi-square NS
Prevalence of moderate to heavy STH infection	17.2% (2.9)	10.7% (2.4)	−6.5%	32.0% (3.5)	17.3% (2.9)	−14.7	Chi-square p<0.001
Mean DMFS in permanent first molars	0.82 (0.12)	1.54 (0.17)	0.72 (0.10)	1.12 (0.16)	1.99 (0.24)	0.87 (0.14)	Student-t NS
Mean PUFA in permanent first molars	0.060 (0.02)	0.137 (0.03)	0.077 (0.02)	0.087 (0.03)	0.220 (0.05)	0.133 (0.03)	Student-t NS P = 0.068

**Table 2:** Mean (± se) data for experimental group after 1 year and the internal non-concurrent control group (grade 2 at baseline examination)

**Indicators**	**Experimental group (1)**	**Internal non-concurrent control group (Group C2)**	**Difference between groups (p-value)**
	n = 168	n = 133	
Mean age	7.56 (0.04)	7.47 (0.04)	Student-t NS
% of boys	50.6% (3.9)	46.6% (4.3)	Chi-square NS
Mean number of siblings	3.12 (0.16)	3.07 (0.16)	Student-t NS
Prevalence of TV ownership	70.4% (3.5)	76.7% (3.7)	Chi-square NS
Mean BMI	14.88 (0.13)	14.86 (0.12)	Student-t NS
Prevalence of children categorized as below normal BMI	27.8% (3.5)	22.6% (3.6)	Chi-square NS
Prevalence of children with moderate to heavy STH infection	10.7% (2.4)	12.4% (2.9%)	Chi-square NS
Mean DMFS of permanent first molars	1.54 (0.18)	1.53 (0.20)	Student-t NS
Mean PUFA of permanent first molars	0.137 (0.03)	0.188 (0.05)	Student-t NS

### Ethical considerations

The study protocol was reviewed and approved by the Institutional Review Board of the Kinaadman Research Center of Xavier University in Cagayan de Oro, Philippines, and it fully complies with the Philippine National Ethical Guidelines for Health Research and the Code of Conduct of the German Development Cooperation GIZ, the financing organization [26,27]. Written consent was obtained from parents or caregivers of children participating in the study. The study is registered with the German Clinical Trial Register DRKS (DRKS00003431) and the WHO system (WHO Universal Trial Number U1111-1126-0718). Based on the Institutional Review Board recommendation, the external concurrent control group of the study also receives a standard intervention (see above) so that no child participating in the study is deprived of potential programme benefits.

## Results

### Reproducibility assessments

The kappa values for inter-examiner reliability for oral examinations were 0.91 and 0.93 for baseline and 1-year data collection, respectively. Sensitivity and specificity for the Kato–Katz test, both at baseline and 1-year data collection, were an average 84.6% sensitivity (confidence interval 68.8–93.6%) and 96.8% specificity (confidence interval 81.5–99.8%) for the diagnosis of STH infections.

### Baseline data collection

A total of 200 children (mean age 6.47 years, 52% male) were examined in the experimental group and 212 children (mean age 6.37 years, 47.1% male) in the external concurrent control group. The baseline data of the experimental cohort and external concurrent control cohort did not show statistically significant differences except for the prevalence of moderate to heavy STH infections (Table 3).

**Table 3:** Mean (± se) baseline data for experimental group (1) and external concurrent control group (C1)

**Indicators**	**Experimental group (Group 1)**	**External concurrent control Group (Group C1)**	**Difference between groups (p-value)**
	n = 200	n = 212	
Mean age	6.47 (0.04)	6.37 (0.04)	Student–t NS
% of boys	52.0% (3.5)	47.1% (3.4)	Chi-square NS
Mean number of siblings	3.34 (0.16)	3.10 (0.13)	Student–t NS
Prevalence of TV ownership	67.5% (3.3)	70.3% (3.1)	Chi-square NS
Mean BMI	14.73 (0.10)	14.64 (0.09)	Student–t NS
Prevalence of children categorized as below normal BMI	28.5% (3.2)	31.6% (3.2)	Chi-square NS
Prevalence of children with moderate to heavy STH infection	17.4% (2.9)	31.1% (3.2)	Chi-square p = 0.0013
Mean DMFS of permanent first molars	0.80 (0.11)	1.16 (0.15)	Student–t NS
Mean PUFA of permanent first molars	0.065 (0.02)	0.090 (0.03)	Student–t NS
Mean dmft primary dentition	7.74 (0.30)	8.27 (0.31)	Student–t NS
Mean pufa primary dentition	3.14 (0.20)	3.11 (0.17)	Student–t NS
Prevalence dmft>0	97.0% (1.2)	97.2% (1.1)	Chi-square NS
Prevalence DMFT>0 for permanent first molars	37.0% (3.4)	42.0% (3.4)	Chi-square NS

### Characteristics of children lost to follow-up

In all, 32 children were lost to follow-up in the experimental group and 39 in the external concurrent control group. More boys dropped out than girls; otherwise, the socio-demographic and clinical parameters of the dropouts were similar to those of the children at baseline in both groups.

### Longitudinal design—comparison between experimental group 1 and external concurrent control group after 1 year

In all, 168 children (mean age 7.56 years, 50.6% male) were examined in the experimental group and 173 in external concurrent control group (mean age 7.39 years, 45.7% male). The mean BMI in experimental group 1 increased whereas it remained unchanged in the control group. The increase in mean BMI was higher (statistically significant) in the experimental group than in the control group (Table 1). The prevalence of children with low BMI in the experimental group decreased whereas it increased in the control group. In both groups, the prevalence of moderate to heavy STH infection decreased, but it was more pronounced in the control group.

Increases in the DMFS (with no changes in the missing or filled index component) and PUFA indexes for permanent first molars over 1 year were lower in the experimental than in the control group (Table 1). The prevented fraction of increases in these indexes was 17% and 42%, respectively. The latter approximates to statistical significance (*p* value = 0.068).

### Cross-sectional design—comparison between experimental group 1 and internal non-concurrent control group

Table 2 presents the data of the 168 children (grade 1) of the experimental group after 1 year and the data of 133 children (grade 2) whose data were collected at baseline. Data comparison between the experimental group 1 and the internal non-concurrent control group did not reveal any statistically significant difference.

## Discussion

### Health outcome assessment of school health programmes

Large-scale, low-cost intervention programmes such as the EHCP have the potential to achieve population-level health effects. Developing and applying data related to public health programmes in low- and middle-income countries is an important basis for improving health. In this context, primary research examining the value of health interventions among poor communities and providing crucial knowledge for informed local policy decisions is of great relevance [28-30]. Field research in low-resource settings often faces challenges related to poor logistics and local resources as well as limited available local staff; thus, research is often organized and undertaken by institutions of the global North. By contrast, in the context of Official Development Assistance, the present research had a strong focus on involving and advancing local research capacities. This study examined a school health programme that is part of a bilateral development project between the Philippines and Germany. The broad participation of local universities and the involvement of Department of Education health have facilitated an increase in the capacity to conduct field research. It is hoped that this will help promote effective health strategies among policymakers in low-resource countries.

It was not intended that this study would produce representative results for the entire child population participating in the EHCP throughout the country. That would have required a complex sampling procedure, a much larger setup, an exponentially higher budget, and a huge management structure. Nevertheless, the various sociodemographic and health-related parameters of the study sample were very similar to those reported in other large-scale or national child surveys in the Philippines [10,31]. The chosen study design provides highly valuable insights related to programme effectiveness under real-life conditions.

### Study design and methodology

Randomized control trials are considered the gold standard for clinical research. However, such a design is often difficult if a large-scale public health programme covers the entire study population or all schools of a province, which is the case with Camiguin Island. There, the authorities did not allow a stepped-wedge randomized control trial since they wanted to introduce the programme simultaneously on the entire island [32]. Randomization of children within a school was impossible and would have been unethical. Since an internal concurrent control school in the same province was not possible, an external concurrent control group was selected despite the problem of inherent selection bias. Schoolchildren in this control group received the traditional health education-based intervention carried out by school health personnel and the deworming programme. In addition, an internal non-concurrent control group was included in the present study. This design allowed for internal comparison and supported the data collected from the concurrent external control group. With regard to the internal non-concurrent control group, evaluation of the outcomes was limited to cross-sectional comparison since it was not a cohort followed over time. The inclusion of an external concurrent control group allowed the assessment of increases in disease parameters.

Generally speaking, cohort studies have a number of advantages but also significant limitations and sources of bias. It has been suggested that four critical areas be examined when assessing the validity of a cohort study [33].

1. Selection bias: Here the essential question is whether intervention and control groups are similar in all important aspects except for the intervention. The children of the experimental cohort were selected on the basis of attending randomly selected schools, whereas the external concurrent control schools were assigned by the regional Department of Education Office. The analysis of sociodemographic data between the experimental and control groups revealed a high degree of conformance—both between each other and in relation to national averages. Moreover, in terms of disease burden, as measured with the different health indicators, the intervention and control cohorts were very similar—except for worm load, which will be discussed in more detail below.

2. Information bias: The study design tried to reduce information bias by limiting the choice of indicators to a few essential ones that were not too complex to measure. The high consistency of examiner results and the constant re-checking through double examinations and duplicate tests indicate a low information bias. The reliability of the caries diagnosis focused on reproducibility with reference to the gold standard examiner; therefore, inter-examiner kappa values were presented. The examiners were blind as to the different groups, although it is probably realistic to assume that the examiners would soon have discovered that the control schools were located in a province where the EHCP did not exist. Appraising the viability, effectiveness, and appropriateness of the screening methods used followed the guidelines of the UK National Screening Committee [34,35].

3. Loss to follow-up: This is a potential source of selection bias and a crucial issue for the validity of cohort studies. Dropout resulted in the loss of 32 children in the experimental group and 39 children in the external control group. It is known that dropout rates are highest for the transition between grades 1 and 2, with a national average of 14.5% of children dropping out of school [36]. The observed loss-to-follow-up rates in the intervention and control groups are thus not surprising. The characteristics of children lost to follow-up in both the experimental and concurrent control group are very similar, which indicates a negligible selection bias.

4. Confounding factors: Socioeconomic factors are usually major confounding factors for health outcomes, with poverty being the strongest determinant of health. The study used a pragmatic approach to assess socioeconomic status by means of proxy measures, such as asking whether there was a TV set at home. Careful questioning is required in this context to avoid information bias. Even the seemingly simple question about the number of siblings can be difficult to answer for children who are not used to differentiating between siblings and cousins and other relatives within an enlarged family. Reassessment of these questions after 1 year helped to trace at least some bias. After 1 year, the mean number of siblings decreased slightly by 0.11 and the number of children whose homes had a TV increased by 3.9%.

### Compliance with study protocol

Compliance with study protocol can be a major confounding factor and needs to be carefully assessed. The results related to deworming are a good example of problems stemming from logistics and lack of compliance to the protocol. Despite careful advance planning and orientation of study staff and participating school personnel, a deworming activity was conducted prior to baseline data collection in the intervention schools. After consultation with the National Institute of Health, it was decided that the data collection be postponed by 6 weeks to give time for partial re-infection. Owing to the complex logistics of the study, it was not possible to delay the baseline data collection any further. This is the reason for the lower prevalence at baseline of moderate to heavy STH infection in the intervention group (17.4%) compared with the external concurrent control group (31.1%). The observed difference of about 15% in the prevalence of moderate to heavy STH infection after deworming is in line with published data [8]. The fact that the reduction in the prevalence of STH infection after 1 year was significantly higher in the external concurrent control group also seems to be related to the deworming of the intervention group weeks before the baseline assessment.

Furthermore, without knowledge of the study team, there was an 8-week delay in starting toothbrushing activities owing to problems with government procurement of supplies.

### Considerations related to BMI

BMI was included in the FITHOS as a derivative health indicator of the EHCP intervention for the following reasons:

• An association between severe caries (PUFA) and low BMI was found in a representative sample of 12-year-old Filipino schoolchildren [37].

• Treatment of severe caries in 5-year-old Filipino children resulted in a significant increase in BMI [38], but the magnitude of the effect caused by using fluoride toothpaste is unknown.

• Medication against STH infection in combination with daily handwashing has a potential beneficial effect on BMI; however, the extent of this combined effect is not known.

We therefore anticipated an effect of the EHCP intervention on BMI, though the overall effect was uncertain. As a result, a power estimation for BMI was not possible.

### One-year analysis and the power of the study

Reduction in the prevalence of moderate to heavy STH infections after 1 year of medication has been found to be around 50% [8]. After 2–3 years, daily school-based brushing with fluoride toothpaste has been reported to result in caries reduction of over 30% [39,40]. This affects the DMF scores, but for the new PUFA score the magnitude of reduction is not yet known. The estimated power for STH and DMFS data in the FITHOS is based on a 4-year study period, and therefore this interim analysis after 1 year provides insufficient power for caries data. However, the main aim of this first paper on the FITHOS is to present the methodology and aspects of the study design.

### Baseline and 1-year results

After 1 year, no differences could be identified in the caries status between the experimental and internal non-concurrent control groups, which may have been due to biological spreading. With longer observation periods of the cohort, the masking effect of biological spreading will gradually disappear, allowing any intervention effect to become apparent. In the longitudinal cohort design, despite the short evaluation period, positive trends became evident: an increase of the mean BMI; a reduction in the prevalence of moderate to heavy STH infection; and a reduction (non-significant) in caries. The confounding effect of the unplanned deworming of the intervention group prior to the baseline data collection will have to be carefully observed with subsequent data collections over following years. So far, the control group design has worked well, with little baseline differences among the groups across all indicators—except for the deworming-related indicators for the aforementioned reasons.

Based on this publication and the emerging data as the study continues, other FITHOS papers will report on progress and elucidate aspects of health equity, effectiveness of oral disease prevention, and quality of life. In combination with a costing study, the health outcome data provide the basis for future cost-effectiveness and cost-benefit analyses. Together with the survey protocol, the key lessons from the FITHOS can help in designing templates for studies in similar countries on ways to adopt the Fit for School concept. Such future multi-country research may contribute to filling gaps in essential knowledge on effective school health programmes worldwide.

## Conclusions

The emphasis of this paper was on presenting the survey methodology and discussing the relevance of this design. The FITHOS has demonstrated that methodology and study design are crucial in collecting viable data on health outcomes where the objective is enhancing programme management, political decision making, advocacy, and donor accountability. This study has demonstrated positive trends in the health impacts of the EHCP after just 1 year of implementation. The key health outcomes thus far are related to handwashing and deworming, resulting in a lower prevalence of moderate to heavy STH infections and an increase in the mean BMI, and to toothbrushing, which has tended to reduce caries. It is hoped that the data over following years will confirm and substantiate these trends by building on the solid survey protocol and functioning logistic support.

## Abbreviations

BMI: Body Mass Index; DMFT: Decayed-, missing-, filled teeth index; DRKS: German clinical Trial register; EHCP: Essential health care programme; FITHOS: Fit for School health outcome study; GIZ: Gesellschaft für Internationale Zusammenarbeit (German Development Cooperation); PUFA: Open pulp, ulceration, fistula and abscess (values for permanent teeth); pufa: Open pulp, ulceration, fistula and abscess (values for primary teeth); STH: Soil-transmitted helminths; WHO: World Health Organization.

## Competing interests

The authors declare that they have no conflict of interest.

## Pre-publication history

The pre-publication history for this paper can be accessed here:

http://www.biomedcentral.com/1471-2458/13/256/prepub
